# Evaluation of the Parkinson’s Remote Interactive Monitoring System in a Clinical Setting: Usability Study

**DOI:** 10.2196/54145

**Published:** 2024-05-24

**Authors:** Bronwyn Bridges, Jake Taylor, John Thomas Weber

**Affiliations:** 1 School of Pharmacy Memorial University St. John's, NL Canada; 2 School of Exercise Science, Physical & Health Education University of Victoria Victoria, BC Canada

**Keywords:** Parkinson disease, usability, remote monitoring, motor examination, movement disorders, thematic analysis, System Usability Scale, mobile phone

## Abstract

**Background:**

The fastest-growing neurological disorder is Parkinson disease (PD), a progressive neurodegenerative disease that affects 10 million people worldwide. PD is typically treated with levodopa, an oral pill taken to increase dopamine levels, and other dopaminergic agonists. As the disease advances, the efficacy of the drug diminishes, necessitating adjustments in treatment dosage according to the patient’s symptoms and disease progression. Therefore, remote monitoring systems that can provide more detailed and accurate information on a patient’s condition regularly are a valuable tool for clinicians and patients to manage their medication. The Parkinson’s Remote Interactive Monitoring System (PRIMS), developed by PragmaClin Research Inc, was designed on the premise that it will be an easy-to-use digital system that can accurately capture motor and nonmotor symptoms of PD remotely.

**Objective:**

We performed a usability evaluation in a simulated clinical environment to assess the ease of use of the PRIMS and determine whether the product offers suitable functionality for users in a clinical setting.

**Methods:**

Participants were recruited from a user sign-up web-based database owned by PragmaClin Research Inc. A total of 11 participants were included in the study based on the following criteria: (1) being diagnosed with PD and (2) not being diagnosed with dementia or any other comorbidities that would make it difficult to complete the PRIMS assessment safely and independently. Patient users completed a questionnaire that is based on the Movement Disorder Society–sponsored revision of the Unified Parkinson’s Disease Rating Scale. Interviews and field notes were analyzed for underlying themes and topics.

**Results:**

In total, 11 people with PD participated in the study (female individuals: n=5, 45%; male individuals: n=6, 55%; age: mean 66.7, SD 7.77 years). Thematic analysis of the observer’s notes revealed 6 central usability issues associated with the PRIMS. These were the following: (1) the automated voice prompts are confusing, (2) the small camera is problematic, (3) the motor test exhibits excessive sensitivity to the participant’s orientation and position in relation to the cameras, (4) the system poses mobility challenges, (5) navigating the system is difficult, and (6) the motor test exhibits inconsistencies and technical issues. Thematic analysis of qualitative interview responses revealed four central themes associated with participants’ perspectives and opinions on the PRIMS, which were (1) admiration of purpose, (2) excessive system sensitivity, (3) video instructions preferred, and (4) written instructions disliked. The average system usability score was calculated to be 69.2 (SD 4.92), which failed to meet the acceptable system usability score of 70.

**Conclusions:**

Although multiple areas of improvement were identified, most of the participants showed an affinity for the overarching objective of the PRIMS. This feedback is being used to upgrade the current PRIMS so that it aligns more with patients’ needs.

## Introduction

### Background

#### Overview

The fastest-growing neurological disorder is Parkinson disease (PD) [1]. PD is a progressive neurodegenerative disease that affects 10 million people worldwide [2]. The incidence and prevalence of PD is rising sharply in countries with aging populations, and in the last 2 decades, the burden of PD has more than doubled, with estimations predicting 1,238,000 cases in North America by 2023 [3,4]. The disease affects the basal nuclei in the central nervous system causing the progressive deterioration of dopaminergic neurons. The loss of these neurons causes motor and nonmotor dysfunctions [5]. Motor system deficits result in symptoms such as tremor, rigidity, bradykinesia, and postural instability [6]. Other symptoms include cognitive problems, gastrointestinal upset, and urinary control issues [7]. Due to these mal effects, PD is linked to morbidity, high economic burden, and decreased quality of life for patients and caregivers. The annual estimated direct and indirect costs of the condition in the United States alone are close to US $52 billion [8]. Neurologists are struggling to manage the increasing prevalence of PD, leading to clinician burnout [9] and lengthy appointment wait times for patients [10]. However, studies show that the management of these symptoms in the early stages of the disease can achieve positive results. In contrast, the consequences of late or faulty diagnoses negatively impact patients and the health care system [11-13].

#### Medication Management

PD is typically treated with levodopa, an oral pill taken to increase dopamine levels, and other dopaminergic agonists. However, as the disease progresses, the effects of the drugs wane. This requires medication dosage adjustments to properly manage symptoms throughout the day [14]. This can be a difficult task for physicians as symptoms are constantly fluctuating and may appear and disappear throughout the day with a hard-to-establish pattern. Some physicians ask their patients to keep diaries where they note the time of day and a description of their symptoms. However, adherence to this method is typically poor and does not provide meaningful information [15]. Therefore, remote monitoring systems that provide more detailed and accurate information on a patient’s condition regularly are a valuable tool for clinicians and patients to manage their medication.

#### Evaluation of PD

The evaluation of PD is commonly performed using clinical rating scales that are essential to the quantification of neurological disorders [16]. These rating scales enable clinicians and researchers to evaluate PD symptoms, progression, treatment efficacy, and disease severity [16,17]. One of the most widely used clinical scales for PD assessment is the Movement Disorder Society–sponsored revision of the Unified Parkinson’s Disease Rating Scale (MDS-UPDRS) [18].

The MDS-UPDRS is a revised form of the original Unified Parkinson’s Disease Rating Scale [17] and incorporates both motor and nonmotor aspects into the assessment. It consists of 65 elements and, on average, requires approximately 30 minutes of administration time. There are four parts to the questionnaire: (1) nonmotor experiences of daily living (13 elements), (2) motor experiences of daily living (13 elements), (3) motor examination (33 elements), and (4) motor complications (6 elements) [18]. Elements are scored from 0 to 4, where 0=normal, 1=slight, 2=mild, 3=moderate, and 4=severe. There are some elements that patients could possibly administer themselves as they are multiple-choice questions asking about personal symptom experience, whereas others are rated by an examiner (typically a neurologist or other clinician) based on observation and physical examination.

While the MDS-UPDRS represents the international gold standard in PD rating scales and has undergone strict validation through clinical studies, it still remains a clinician-based scale; this means that a clinician assigns a score based on their own personal qualitative observations of a patient. Therefore, the assessments are often subjective and biased to the examiner’s skill and knowledge. The assessments will also vary from one examiner to the other in this way [19-21]. Studies have shown that there is variability between assessments conducted by nurses and neurologists [22,23]. In these situations, it is difficult to compare and interpret the scores, as they may differ based on a patient’s condition or simply due to the clinician performing the assessment. The MDS-UPDRS is also time consuming for clinicians. It requires approximately 30 minutes of an examiner’s time, which makes it impractical for routine practice [18]. Examiners must also be highly trained to improve the validity of the scores. Many of the elements in the MDS-UPDRS must be completed by a patient themselves, which adds to the time burden of the questionnaire when performed in a clinician’s office. The typical assessment performed in a clinical setting rarely assesses a patient’s day-to-day symptoms, which usually vary over time, and only captures a snapshot of an individual’s condition at the moment of their appointment [24]. Patients also typically have long wait times in between their appointments, which makes it difficult to remember their symptoms since their last visit [10]. This way, medical decisions are now influenced by recall bias and patient attitudes instead of by reliable patient data. In addition, it is an inconvenience for patients to travel to clinics due to transportation, long commutes, and their conditions, especially if they are in the advanced stages of PD. Therefore, there is a need for objective, accurate, and reliable assessment tools that can help increase the chance of effective treatment. These could aid patients with their disease management, thus cutting down on health care costs [25].

### Digital Health Technologies and the Parkinson’s Remote Interactive Monitoring System

An emerging solution to some access to health care issues are video-based visits. These bring care directly into a patient’s home, which improves access in a patient-centered manner and minimizes the burden on people with PD and their caregivers [26]. In addition, due to the largely visual nature of a PD examination, it tends to work well in a video-based visit. Studies have shown that web-based appointments with neurologists are feasible and valuable [27,28]. It has also been shown that a modified version of the MDS-UPDRS motor examination (excluding the test of rigidity and postural stability) can be successfully administered remotely [29]. However, as virtual visits still require a clinician’s time, they still only provide a brief snapshot of a patient’s condition. There need to be other methods of assessing PD without occupying already overburdened clinicians.

Digital health technologies that alleviate the need for medical professionals to assess disease progression have been on the rise. These technologies offer possibilities for self-assessment and improved health care [30]. Some of the technologies developed for PD include wearable sensors and mobile apps. These devices have been used extensively to monitor motor symptoms and complications of people with PD in their home environments [31]. These wearable sensors and mobile apps can accurately track the progression of PD [32-34] and other neurological conditions [35]. Examples of these devices on the market are the Global Kinetics Corporation’s Personal KinetiGraph Watch [36,37] and Rune Labs’ StrivePD mobile app [38]. APDM Wearable Technologies has also developed multiple sensors that can accurately monitor tremor and dyskinesia symptoms of PD that have been used in many clinical studies [32,34,39,40]. Other wearables that collect contextual data include DynaPort MiniMod Hybrid (a sensor worn on the lower back), Shimmer (records gait), SENSE-PARK (records walking, hypokinesia, dyskinesia, and sleep), activPAL, and StepWatch (gait and basic movement parameters) [20,41-45]. The problem is that these technologies only generate a small amount of patient data (mainly tremors and other motor symptoms, moods, and sleep characteristics). Therefore, although these devices provide an objective means of tracking PD characteristics, they do not provide a complete assessment of the condition. Wearable sensors also have inherent risks [46] and do not follow the gold standard clinical scales such as the MDS-UPDRS. These risks encompass potential interference with the daily activities of patients with PD, impacting their natural movements and behaviors. In addition, behavioral modifications stemming from the feedback provided by sensors can yield both positive and negative outcomes. On the positive side, such modifications may encourage beneficial lifestyle changes and provide meaningful data. However, as a downside, they may also contribute to increased anxiety and foster a dependency on the wearable device [46].

To address the need for reliable tools to objectively assess PD symptoms that do not require a clinician’s involvement, the Parkinson’s Remote Interactive Monitoring System (PRIMS) was developed. The PRIMS is a digitized version of the MDS-UPDRS in the form of a desktop application that people with PD can complete themselves. This way, the PRIMS provides a complete picture of PD assessment via its capacity to comprehensively measure both motor and nonmotor symptoms without the need for wearable sensors and its potential to serve as a valuable tool in a clinical or home setting. If validated through further investigation, the PRIMS has the potential of delivering a standard in PD assessment. The PRIMS also has the potential to be valuable in a home setting, offering a user-operated system capable of capturing a significant portion of the MDS-UPDRS (considered the gold standard). The system provides patients with a means of tracking their condition remotely and offers clinicians reliable data for better medication management. This comprehensive approach enhances understanding and facilitates more effective monitoring of the progression and individual symptoms of a patient.

### Usability Testing

The development of any system that is used by patients and clinicians for the management of biomedical data should always involve usability evaluations, which aim to understand whether such a product is easy to use and has the appropriate functionality for the users. *Usability* is a term used to define how easily people can use a tool or object to accomplish a specific task [47,48]. In this way, when developing interfaces, it is imperative that they can be learned quickly and are easy to navigate. The system’s layout should avoid and manage operational errors efficiently and provide users with appropriate feedback [47]. Usability must also address user satisfaction and provide solutions to the problem that the system was designed to solve [49]. A common method of assessing usability is the System Usability Scale (SUS). The SUS has been used in multiple studies, such as the evaluation of a mobile app for people with PD [50]. Structured interviews are common practice for these types of studies [51]. Field notes can also be a valuable tool for qualitative researchers to collect and analyze [52]. Observational notes can capture information such as the nonverbal reactions of users while they interact with the system. This study used multiple methods to assess the usability of the PRIMS.

### Study Objectives

This study aimed to assess the functionality, usability, and user experience aspects of the most recent version of the PRIMS in a clinical setting from the perspectives of people with PD. Use issues identified in this study will guide designers in creating a more effective commercial product. Using multiple methods, including interviews and field notes along with SUS surveys, we evaluated the user experience of the PRIMS.

## Methods

### Participants

Participants were recruited from a user sign-up web-based database owned by PragmaClin Research Inc. The study was also advertised by the Parkinson Society Newfoundland and Labrador on their weekly newsletter. Interested participants who contacted us were given a questionnaire that determined their eligibility for the study. The inclusion criteria for study participation were the following: (1) being diagnosed with PD and (2) not being diagnosed with dementia or any other comorbidities that would make it difficult to complete the PRIMS assessment safely and independently. Participants were recruited on a first come, first served basis. Informed consent was obtained from all participants via a web-based consent form emailed to them before study completion. Paper copies were also available to participants at the time of their scheduled session.

### Ethical Considerations

This study received ethics approval from the National Research Council of Canada Institutional Review Board (protocol 2021-137). Informed consent was obtained, and the possible consequences of the study were explained. All data were deidentified. No compensation was provided to participants.

### Description of the PRIMS

**Figure 1:**
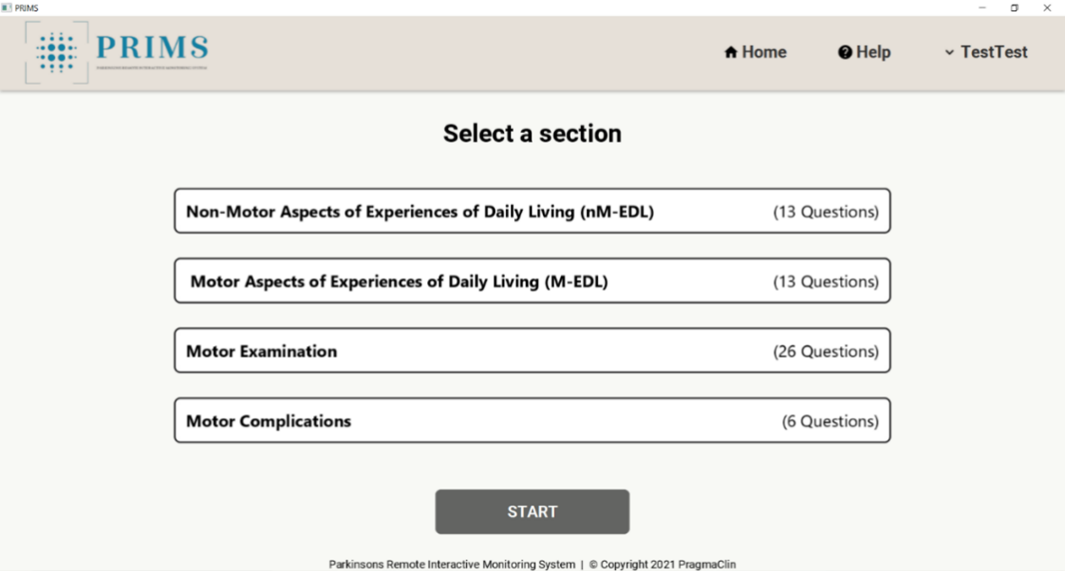
The 4 sections of the Parkinson’s Remote Interactive Monitoring System (PRIMS) questionnaire based on the Movement Disorder Society–sponsored revision of the Unified Parkinson’s Disease Rating Scale.

**Figure 2:**
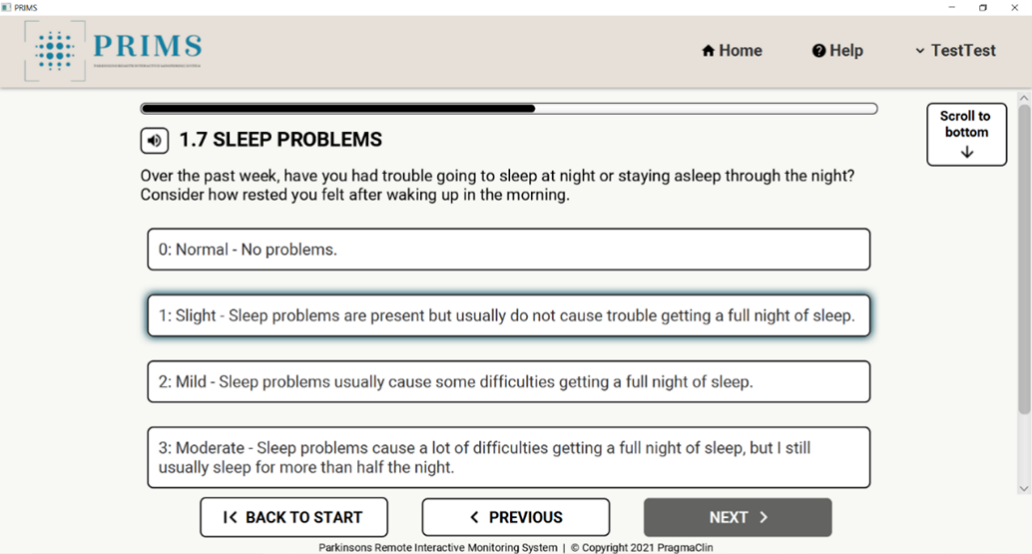
Example multiple-choice question.

**Figure 3:**
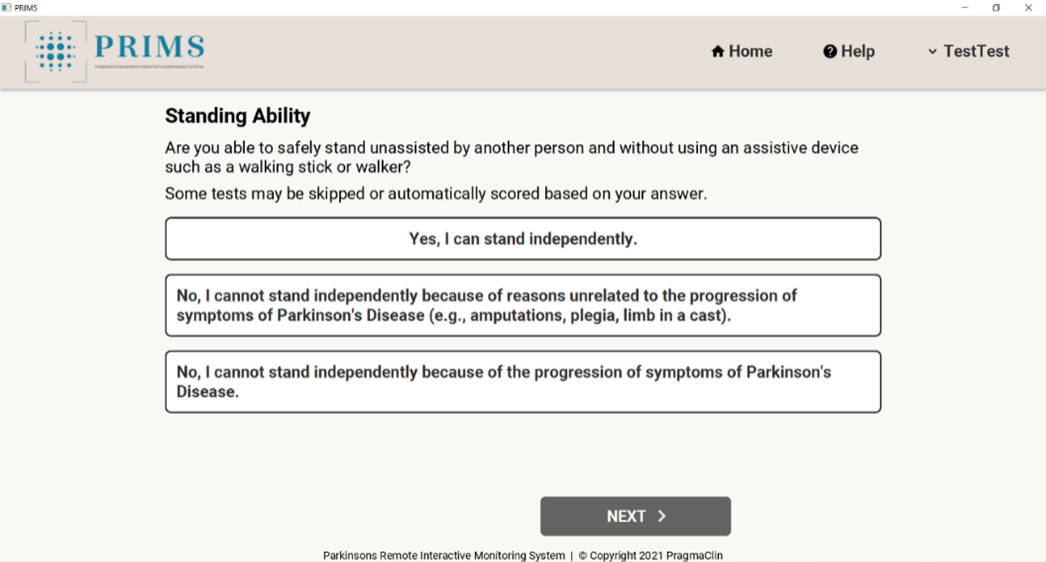
Multiple-choice question assessing an individual’s ability to stand.

**Figure 4:**
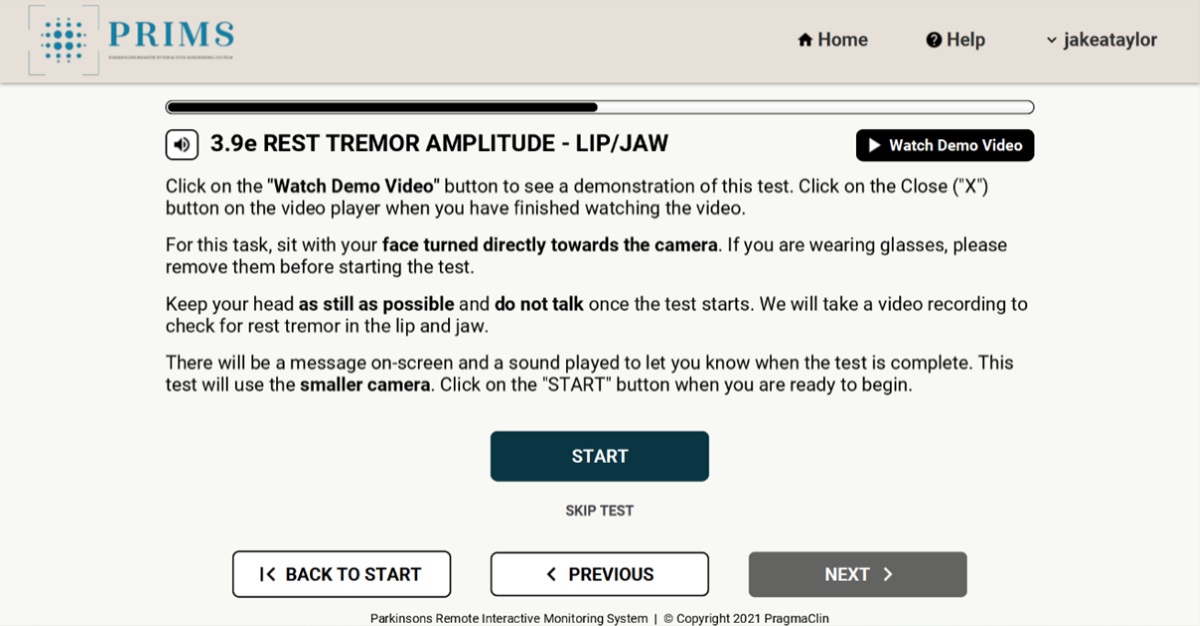
Example of a motor task page.

**Figure 5:**
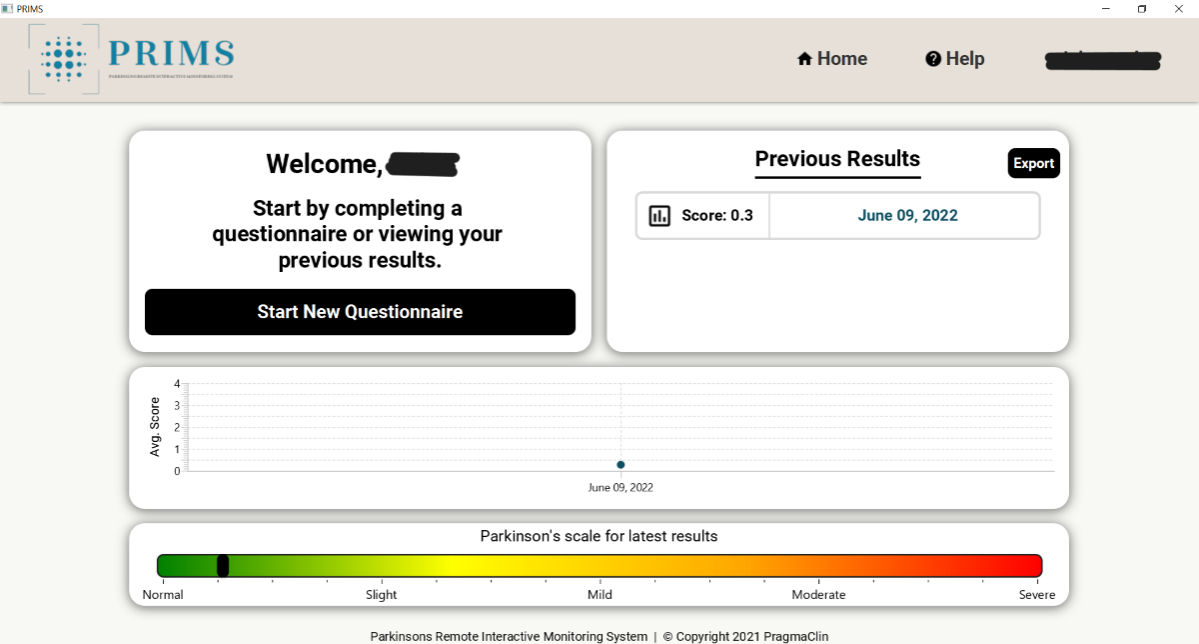
Screenshot of the Parkinson’s Remote Interactive Monitoring System (PRIMS) home page.

The PRIMS was developed by PragmaClin Research Inc and was designed on the premise that it will be an easy-to-use digital system that can accurately quantify motor and nonmotor symptoms of PD remotely. The PRIMS has the capability to interact with patients in real time, delivering results promptly through a dedicated patient dashboard. Patients can access their dashboard by logging into the web-based platform to see a history of their assessments. Patient users complete a questionnaire that is based on the MDS-UPDRS. The questionnaire comprises 4 sections shown in Figure 1. Of these sections, 3 are multiple-choice questions based on daily living experiences; an example is shown in Figure 2, and there is also a motor examination where users perform tasks similar to those outlined in the MDS-UPDRS. Data are captured via 2 depth cameras (Intel models D435 and D455) that track a patient’s movement in 3D. Before completing the motor examination, there is a series of ability questions that determine whether the user can safely perform all motor tasks; an example is shown in Figure 3. Motor tasks are explained in written form on the screen along with a demonstration video that presents users with a visual walk-through of the movement; an example is shown in Figure 4. The intelligent software scores each motor task based on the same parameters as the MDS-UPDRS. However, it is important to note that the system’s scoring has not yet been validated. After users complete the 4 sections, a participant’s responses are analyzed to put an individual on a PD rating scale from 0 to 4 (0=normal, 1=slight, 2=mild, 3=moderate, and 4=severe). A summary of a user’s scores is presented on the home page, which can be seen in Figure 5. The survey was intentionally crafted and edited from the original MDS-UPDRS to use layperson language for easy comprehension. Although some technical terms appeared in titles or examples, they were not essential for answering questions or comprehending instructions.

### Equipment

The PRIMS was run on a Dell G15 laptop computer, and Intel RealSense D435 (small—stand-alone mini tripod beside the laptop) and D455 (large—mounted on the computer) depth cameras were used. Participants sat on a contemporary midback task office chair with wheels for the entire questionnaire and had the option of using a Kensington Pro Fit wireless computer mouse. Interviews were recorded using a HyperX SoloCast stand-alone microphone. Audacity (Muse Group) was used as an audio recording and processing software. The computer-assisted qualitative coding software Delve (Twenty to Nine) was used for thematic analysis. All audio files were transcribed into Word (Microsoft Corp) before being uploaded to Delve. Qualtrics XM (Qualtrics International Inc) was used to administer the SUS survey and the virtual consent form.

### Usability Testing Protocol and Procedure

Usability testing occurred at PragmaClin Research Inc’s office site and was carried out by a trained research assistant (RA). Before the start of testing, the RA explained the study objective and research protocol to the participants. The RA also provided detailed information about the test procedures and described the purpose of the PRIMS. The example script is provided in Multimedia Appendix 1. As the research team was interested in how participants interact with the system when there is no one present to assist them, the RA was not allowed to help unless deemed necessary. The necessity to intervene in the form of helpful hints or prompts or manipulating the system was operationally defined as any circumstance in which the participant was unable to progress through the system without aid. An observer was present during the entire session. All participants used the PRIMS only once and reported user experience from this single use.

After the participants gave informed consent, they were instructed to start using the PRIMS. The initial PRIMS developed by PragmaClin Research Inc was used during the usability testing. While participants worked their way through the assessment, they were encouraged to vocalize any confusion or ask any questions.

Data were recorded in the form of field notes, a short qualitative interview, and an SUS survey administered to participants after they completed the PRIMS questionnaire. Recorded data were used to identify a set of usability issues.

### Field Notes

Structured observation was used to analyze the users’ interactions with the system. During the session, the RA was instructed to observe and note any issues that arose along with any user critiques, comments, questions, difficulties, or observations about their interaction with the system. Thematic analysis was performed on these notes in Delve. Usability issues were identified via this method of analysis of the written notes.

### Qualitative Interviews

After participants were finished with the PRIMS, they completed a short qualitative interview. The interview consisted of six questions: (1) What things did you like most about the PRIMS? (2) What things did you like least about the PRIMS? (3) Were there things about the PRIMS that you found confusing or frustrating? (4) What would you like to change about the PRIMS? (5) Are there any features that you would like to see added to the PRIMS? (6) Do you have any overall comments on the PRIMS?

Audio was transcribed and analyzed in the qualitative coding software Delve. Thematic analysis was performed following the framework by Braun and Clarke [53]. The themes were discussed, reviewed, and interpreted by the research team.

### SUS Survey

After the interview was finished, participants completed a short-answer quantitative questionnaire following the standard SUS approach devised by Lewis and Sauro [54] in 2018. A copy of the survey is provided in Multimedia Appendix 2.

SUS scores were output in Qualtrics XM and then analyzed in Microsoft Excel. To calculate the SUS score, first the score contributions from each item (question) were summed. Each item’s score contribution ranged from 0 to 4. For items 1, 3, 5, 7, and 9, the score contribution was the scale position minus 1. For items 2, 4, 6, 8, and 10, the contribution was 5 minus the scale position. We multiplied the sum of the scores by 2.5 to obtain the overall value of the SUS score. SUS scores have a range of 0 to 100. SUS scores of >70 points are considered acceptable usability (according to various other usability studies), and scores of >85 are regarded as excellent usability [55]. The curved grading scale by Lewis and Sauro [54] that was used to interpret the scores from the SUS is provided in Multimedia Appendix 3.

## Results

### Participants

A total of 11 people with PD participated in our study (female individuals: n=5, 45%; male individuals: n=6, 55%; age: mean 66.7, SD 7.77 years). Data from 91% (10/11) of the participants were fully analyzed as 1 user dropped out during the testing session. The 10 participants took, on average, 67.7 (SD 16.4) minutes to complete the motor examination and 84.2 (SD 23.3) minutes to complete the entire PRIMS questionnaire. Participants skipped 2.9 (SD 1.97) motor tests on average (total of 29 skipped tests). Table 1 shows which tests were skipped the most and how many times they were skipped.

**Table 1:** Number of times each motor test was skipped (tests ranked from the highest to lowest number of skips).

Motor test	Skipped tests, n
3.15	12
3.14	6
3.13	6
3.3	2
3.8	1
3.9e	1
3.10	1
3.1	0
3.2	0
3.4 and 3.5	0
3.6	0
3.7	0
3.9a-d	0
3.11	0
3.12	0

### Field Notes

Thematic analysis of the observer’s notes revealed 6 central usability problems associated with the PRIMS. These were the following: (1) automated voice prompts are confusing, (2) the small camera is problematic, (3) the motor test exhibits excessive sensitivity to the participant’s orientation and position in relation to the cameras, (4) the system poses mobility challenges, (5) navigating the system is difficult, and (6) the motor test exhibits inconsistencies and technical issues.

#### Automated Voice Prompts Are Confusing

The RA noted on multiple occasions that the participants found the automated voice prompts to be confusing. Frequently, participants would begin the test and align themselves in a good position; however, when presented with audio prompts such as “make sure hand is not tilted” or “adjust hand position or angle,” users would move in all directions:

The automated voice prompts were confusing and making it difficult. Even though P8 was turned in the right direction, the system still prompted him that he was turned the wrong way.

A lot of automated verbal instructions were getting fired at P8, this made the task confusing and frustrating since they will be in the correct position and the software tells them wrong direction, turn right, etc.

The prompt “Adjust hand position or angle” was especially frustrating and confusing to participants. Many verbalized their confusion with the statement. On multiple occasions, the RA noted that participants were becoming frustrated with the motor tests when the audio prompts began giving them instructions:

The prompt—no hand detected—is vague and confusing. P6 was in a good position, with their hand fully in view...Plus, P6 did not move and then immediately after there is a prompt saying—no hand detected...the audio prompts confused and frustrated P6.

The RA also noted that some participants complained that the voice commands were authoritative and unfriendly:

...get in position and stand still—is a little authoritative! P8 mentioned this, participant did not like the automated voice instructions, said it needs to be more comforting / friendly. The automated voice prompts were authoritative P3 mentioned.

Comments also often noted the repetitiveness of the automated voice prompts. They were repeated too often and in bizarre patterns, which caused confusion and frustration among users:

“don’t move body in good position” was constantly repeating...was repeated ~5 times over, and the test wouldn’t start.

“Make sure hand is fully in view” is repeated even when the hand is fully in view, The system was prompting repeatedly “make sure hand is not tilted,” motor test was very particular on positioning here. This made hand and body measurements very difficult for P2. They were moving their hand in all directions trying to figure out what tilted meant.

Overall, participants found the automated voice prompts to be vague and irritating, as they rarely provided useful corrective feedback.

#### The Small Camera Is Problematic

The RA frequently noted problems associated with the tests that used the small camera or issues directly related to the small camera itself. The narrower field of view was an issue for multiple tests; participants frequently moved out of the camera view midway through a task:

Finger to nose movements were difficult for P7, they had a hard time staying in the cameraview. They would be prompted that they are in a good position, then move out of the cameraview when performing their finger to nose movements. Hand moves out of frame...when the participant does hand rotations...small camera issue. P4 had great difficulty staying in the cameraview for these hand rotation tasks.

The system would frequently tell users that they were in a good position, but when they started performing the task, they would move out of the camera view. The setup of the small camera also created problems for users. Users had to manipulate and adjust the small camera on the tripod to align themselves in a proper position. Frequently, this would lead to participants becoming uncomfortable due to the poor ergonomics of the system:

Face measurements using the small camera were not comfortable for P4. Small camera moved P4 into an uncomfortable position.

The fact that P3 had to look at the camera for the test but look at the screen to get into position was causing difficulties.

Getting into position was difficult for P9. Again, ergonomics is poor here for performing the test on two different sides when the camera is on one side.

The tests that asked users to adjust the small camera were especially problematic. The RA frequently noted that people who pulled the cords out when laying the camera down for the final 2 tests did the following:

P2 moved the tripod/small camera, it was sloppy and difficult to work with. P2 pulled the cord out while adjusting the small camera.

P11 unplugged the camera when they moved it for 3.15...issue with adjusting camera.

Overall, the RA noted far more issues with the tests that used the small camera compared to those that used the larger camera.

#### The Motor Test Is Excessively Sensitive

It was clear from the RA’s notes that participants had difficulty getting into what the system would consider to be valid positions to score during the motor examination. At times, the system would prompt users to stay still:

P9 had difficulty with hand measurements, they were unable to hold their hand still (issue since the software is for PwP). System is far too particular. The hand and body measurements require users to stay still to capture the measurement; P11 had great difficulty with this. System is far too particular on positioning here, which is just not feasible for those with PD.

The system in its current state is very sensitive, which posed challenges for users:

The software is too picky on the positioning, participant’s dyskinesia made it very difficult to stay in position, when the software told P7 that they were in a good position, they only had to move very slightly for the software to tell them that they needed to “adjust hand position or angle.”

A slight tilt is all it took for P8 to move out of position. The system was very particular on the positioning of the limb.

The RA noted on multiple occasions that the system would repeat certain prompts when users were close to getting into the correct position:

“Don’t move, hand is in good position” repeats a lot when P6 was “on the edge” of a good position. And when you start rotating your hand “no hand detected.”

“Don’t move, hand is in good position” repeated a lot when P5 was “on the edge” of being in a good position.

The system is far too sensitive, “Don’t move face is in good position” kept repeating even though P3’s face was in a good position. The motor test is too picky, its needs to be able to get the measurements from a broader range of places.

Overall, it was clear from the recorded field notes that the motor examination was difficult for users:

3.14, and 3.15 P9 had a lot of difficulty keeping their hand in the correct position, P8 had difficulties getting their hand perfectly parallel to the camera face, The fact that P3 had to look at the camera for the test but look at the screen to get into position was causing difficulties.

#### The System Poses Mobility Challenges

The setup of the PRIMS posed various mobility challenges for users. The RA noted on multiple occasions that users felt that the chair and constant movement of users were both issues. In its current state, the PRIMS requires participants to move back and forth from the computer to go from one task to another. The RA noted on several occasions that this posed a challenge for users:

Moving back and forth from the computer was difficult...the system requires too much movement of the chair, and to and from the computer, P7 vocalized this.

This, coupled with the fact that users are constantly moving the chair in and out of the camera view, made the motor examination tiring for participants:

There is constant movement to and from the system that was tiring P8.

The chair was difficult to work around due to the nature of the test; the RA noted repeatedly that participants failed to complete tests due to the chair obstructing the camera view:

...chair was in cameraview during these tests, posed an issue, was also difficult for participants to work around it.

Some participants were also obstructed by the legs of the chair:

P5 needed to work around the chair legs for the arising from chair task, they vocalized this. The legs of the chair were in the way.

Multiple participants also pointed out that they thought the chair was a safety issue:

The chair with wheels concerned P2. They thought it was a safety issue.

P3 mentioned a few times that people with PD are told NOT to use chairs with wheels.

For safety reasons, the chair with wheels is a huge problem, P7 vocalized this.

Overall, the maneuvering required to complete the motor examination was an issue for users.

#### Navigating the System Is Difficult

There were frequent comments made by the RA on navigational issues users had while working through the system. Many participants had issues with the required amount of scrolling:

Scrolling is too difficult. a bigger screen would allow the entire survey to fit on one screen. P5 found the scrolling to be a challenge right away.

P9 again demonstrated issues with scrolling and navigation, they had trouble scrolling to the bottom of the screen to select next on multiple tasks.

Some users even found that they made mistakes due to the need to scroll to the bottom of the page:

P7 found that the scrolling led to mistakes. Choices they didn’t mean to select.

The RA also noted that the amount of clicking was an issue, specifically accurate clicking:

Skip test button is too small. Navigation issue, P9 had trouble clicking it due to dyskinesia, since it was so small.

P7 had trouble closing the demo videos. Too much accurate clicking / total clicking required.

Users also had issues with the computer mouse and mentioned that a touch screen interface would be preferred:

P9 had significant difficulties with the mouse. They said that they would prefer a touch screen.

P2 didn’t like using the mouse...Stated right away that they wanted to use a touch screen.

The test window and demonstration video window also caused issues for users. Demonstration videos would frequently open in inconsistent sizes and locations of the screen, making them difficult to close:

Demo videos were opening in small windows at the top of the screen. Made them difficult to close and to watch.

The test window did not show a married image of the person, which made it confusing to get into position:

Screen being non-mirrored is an issue. P5 had trouble moving into position because of this.

The RA also noted that, as the software was not entirely full screen, users would frequently open other programs by accidentally clicking on the bottom task bar:

Full screen should eliminate the lower task bar (desktop), P4 ended up clicking things below or bringing up the news.

Overall, users had difficulty navigating the system to progress.

#### The Motor Test Exhibits Technical Issues

There were frequent notes made on the system not operating correctly. Users would perform tests correctly or be told that they were in a good position yet would still be asked to try again as the system did not capture enough valid measurements to score:

Hand movements test stated that there were not enough valid measurements to score, after the participant did everything correctly.

There were also occurrences in which users would perform tasks incorrectly and the test would still function:

Postural stability test worked even though the participant was not in the correct position at all.

The foot tapping test ran even though P3 tapped the wrong foot, the system still gave him a score. They performed the measurement incorrectly, yet the system still considered it to be valid.

This would lead to confusion among users as they would go through tests being told that they were in a good position without any other corrective feedback only to be asked to try again:

P6 performed the test correctly without any prompts to change position yet the system still prompted them to try again. The test will prompt people to start walking, and will run through without any corrective feedback, but may still state that there were not enough valid measurements to score.

Some tests also tended to shut off very early and inconsistently. Other tests would often produce nonsense automated voice prompts:

...while performing the finger to nose movements: the audio prompt “multiple hands detected” was repeated even though there was only one hand in the camera view.

Overall, the motor test presented frequent glitches causing usability trouble for participants.

### Qualitative Interviews

Thematic analysis of qualitative interview responses revealed 4 central themes associated with participants’ opinions on the PRIMS. These were (1) admiration of purpose, (2) excessive system sensitivity, (3) video instructions preferred, and (4) written instructions disliked.

#### Admiration of Purpose

Most of the participants showed an affinity for the overarching objective of the PRIMS:

I know what the main objective is, and I applaud that, that is a good objective.

Participant 8

They were excited about the system being available to people with PD:

I think it is awesome that people will have access to this.

Participant 11

I just like the fact that this is available for people.

Participant 11

I am sure a lot of people would be thrilled to have this at their doctor’s office.

Participant 11

The intended purpose of the PRIMS was also well received and understood. Participants liked the idea that this will give their physicians a better view of their condition and support their ability to do their job:

Doctors often don’t have a lot of time to do the examination in depth. My in-person examinations with my Neurologist are very fleeting, and scratching the surface in my view, but if that is the norm, and my Neurologist got a good reputation, then something like this would be very very helpful.

Participant 8

I like how then your doctor would have a better idea of what you are doing really, rather than based on that little scope of time kind of thing. Yea...that would be good.

Participant 6

I like that it can be used for long distance. And in our new post covid medical system, we need to free up time for our doctors.

Participant 3

Overall, participants admired the system and what it is trying to achieve and were excited to see the finished product in the future:

I think it would be worthwhile, if it was something that was worked out, if all the bugs and stuff were worked out it could be used as a tool...

Participant 4

Overall, I think it’s a pretty good system. I think it will help patients or people.

Participant 1

#### Excessive System Sensitivity

Most participants found that the motor examination was very sensitive, which made it difficult to get into the proper position for the tests:

Yea like I say it’s too sensitive, cause we have Parkinson’s, and most people, you know you can be [shaky] and there’s no way you are going to be able to stop it [tremors].

Participant 2

Users also found it frustrating and time-consuming:

Well I didn’t like how it kept telling me that my hand is in a good position and then it’s not or they don’t detect it or those kinds of things, it can get a bit frustrating.

Participant 1

The only thing is the actual working of it in those couple of times where no matter what I did or didn’t do, everything was as still as I could make it and it says you are fine then it says nope you have to start again...oh my gentle god...maybe it’s just too sensitive or something.

Participant 2

It is time consuming too.

Participant 11

It is long and...you know...as Parkinson’s patients you get tired easily.

Participant 6

The frustration seemed to stem from the fact that the system was very particular on how it wanted users to be positioned. Participants had the greatest trouble with the hand movements and tasks that required users to stay still:

The ones we have the most difficulty with are the hand.

Participant 8

Because holding still is a challenge for some people with Parkinson’s...some people have tremor, and some don’t. For those that do, holding still is a real challenge.

Participant 11

Overall, users found that the system was difficult to use in its current state due to its sensitivity:

I think that that [PRIMS] would be difficult for some people...unless you had extensive training.

Participant 4

#### Video Instructions Preferred

Users found that the demonstration videos were far more helpful, and much less confusing, than the written instructions:

The video was a good tool because we have a lot of brain fog, and reading can be confusing and looking at the video makes things much easier.

Participant 3

...you are able to see a video of the man actually doing what you are supposed to do you know is quite helpful too I thought.

Participant 8

There are a lot of words there, in the instructions, again I think if you had it in bullet form maybe. It’s a lot easier to watch the video.

Participant 1

The video was good to show how to do the testing.

Participant 1

Users suggested more video instructions and less written instructions and even suggested an introduction video outlining what the system entails:

Instead of just jumping right in there, if you had, well I guess it would be a video, but if you had a synopsis of what the testing involved. Maybe if we had a 10-minute video overlooking the whole test at first.

Participant 1

Overall, the videos were one of the most liked aspects of the entire system:

The things that I thought worked best were the videos.

Participant 5

I like how there is a video...watching what they do is much more clear.

Participant 11

#### Written Instructions Disliked

Many participants found that the written instructions were vague and confusing:

Some of the tests I found confusing, but again that was the written instructions that were somewhat confusing.

Participant 5

The instructions were really kind of vague.

Participant 1

Multiple users stated that these instructions were annoying and far too wordy:

Too much instruction, yea, but I know you have to have the instruction down, but it was a lot of reading.

Participant 1

I would say all the text that open on the screen, yea it’s like going to a presentation...mostly just the instructions, I mean you’re asking me, in my gut, kind of what I found to be annoying about it...and...the text was annoying.

Participant 7

Participants suggested that more concise bullet-point instructions would be preferred over written paragraphs:

I think the instructions was too many...If it was concise and shorter instructions, I think it would make it a little better.

Participant 1

### SUS Survey

The average SUS score was calculated to be 69.2, which corresponds to a C on our curved grading scale [54]. The PRIMS failed to meet the acceptable SUS score of 70.

## Discussion

### Principal Findings

We conducted a multiple methods study to assess the usability, functionality, and user experience of the PRIMS. Thematic analysis of interview transcripts and field notes revealed multiple themes and usability issues, respectively, that describe the tested product. An SUS survey also gave us a key objective insight into the system and its user experience.

One of the key findings of this study was that video instructions were preferred over written instructions. Thematic analysis of interview transcripts revealed these 2 themes (*written instructions disliked* and *video instructions preferred*). Multiple participants stated that the video instructions were much less confusing and much more informative than the on-screen text. The written instructions were designed to give all the necessary information to complete the task. This may have resulted in users feeling unmotivated to read the entire set of instructions as there was an intimidating amount of text present on-screen. Other investigations comparing video to written instructions have found similar results. Cosford et al [56] evaluated the effectiveness of video and handout instructions during a veterinary student examination. Their findings revealed that students using video instructions achieved notably higher scores, suggesting a better understanding of the tasks compared to those using handouts [56]. Shah and Gupta [57] found that video instructions were significantly more effective than written instructions in teaching inhaler use technique. Video instructions provide both a visual and audio description of each task, which can make the instructions both clearer and less time-consuming.

Another principal finding was that the PRIMS motor examination was too sensitive and particular on users’ body positions during the tests. Thematic analysis of field notes and interview transcripts unveiled 2 areas of issues, namely, system sensitivity and the motor test’s positioning specificity, which exhibited alignment in their respective scopes. To quantify each motor task performed during the PRIMS questionnaire, the depth cameras would require participants to be oriented in a “good position.” From the RA’s observational notes and the interview transcripts, it was clear that the system asked too much of users, which led to frustration and difficulties. Systems designed for those with movement disorders must be accommodating to their needs. The PRIMS, in its current state, asks users to stay still in certain situations and adopt specific and uncomfortable positions to score their movements. Future versions of the PRIMS will need to address this in their design and implementation.

Another theme revealed from analysis of field notes was that the automated voice prompts that are used during each motor test are confusing to participants (*automated voice prompts are confusing*). The prompts would also do more harm than good when it came to helping participants align themselves in the correct position for each motor test. It was noted that users tended to move in all directions in response to the automated voice. This could be due to the vague nature of the instructions provided by the prompts. They also led to frustration and confusion, making them an ineffective tool to guide users through the tests. Some users even stated that they found the automated voice to be authoritative and unfriendly, which only increased their frustration with the system. The consensus of this key finding was that these automated prompts did not provide any useful corrective feedback and only led to confusion and frustration among participants. Mays et al [58] delved into how people in the United States perceive automated communication, such as interactive voice response systems. They found that older respondents especially did not enjoy the automated voice system and exhibited greater levels of frustration toward it [58]. Most people with PD are older individuals; therefore, it would be best practice to tailor the system’s instructions and prompts to their typical preferences.

**Figure 6:**
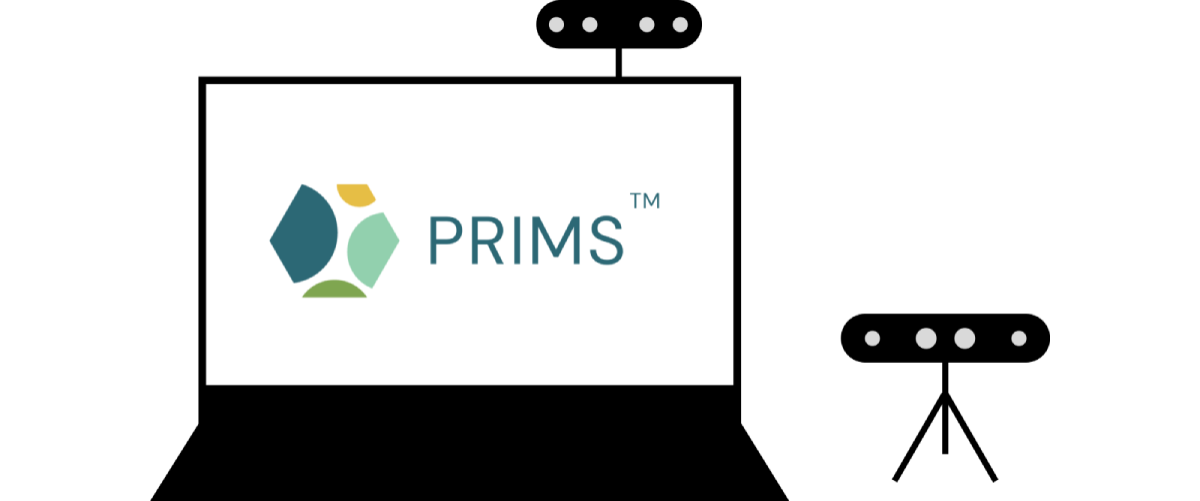
Diagram of hardware setup showing the position of the laptop and 2 depth cameras.

Our next key finding is that the small camera tended to cause more problems for users than the large camera. Thematic analysis performed on field notes revealed this theme (*the small camera is problematic*). The smaller-depth camera (Intel D435) has a narrower field of view and was primarily used for the hand movement tests during the motor examination. A common problem that users faced was staying within the camera view for these tests. As the RA noted on several occasions, it was common for participants to have difficulties with the narrow view. The position of the small camera also caused trouble for users. Unlike the larger camera, the smaller camera is placed on a tripod on either side of the computer (Figure 6). It was noted frequently in the field notes that users had to manipulate and adjust this camera to align themselves in the proper position. The placement of the camera also negatively impacted the system’s ergonomics. In addition, for tests 3.14 and 3.15, users were prompted to flip the tripod down so that the camera was facing the ceiling. Frequently, users accidentally disconnected the cords from the camera when moving it. In general, a setup in which users do not have to adjust any equipment would be preferential. To our knowledge, there is no direct study to compare this finding to.

Another principal finding of our thematic analysis was that the PRIMS has associated mobility challenges for users. A big issue that the design of the system has is the constant movement to and from the computer to go from one task to another. At times when the users’ feet or whole body had to be visible to the camera, participants would have to move backward until this was the case. To move onto the next task, users would have to return to the laptop to select it. This way, users are constantly moving to and from the computer and frequently being obstructed by the chair. Several users even pointed out that they felt as though others would have problems with the back-and-forth nature of the system. When designing systems for those with movement disorders, it is important to consider the user experience as a whole.

Thematic analysis of field notes also revealed that users had difficulties navigating the system (*navigating the system is difficult*). In its tested state, the PRIMS was operated using a standard computer mouse or laptop touchpad (depending on user preference). Navigating the system using either of these tools caused difficulty among participants. Scrolling or clicking to move in between sections of the questionnaire was frequently noted as a challenge for users and even led to mistakes in some circumstances. The accuracy of the clicking to move through the system was also a big issue and would often lead to opening other applications or closing the PRIMS software. It is important to remember that, when designing systems for those with movement disorders, there must be special considerations taken. A viable option could be a touch screen device featuring prominently sized buttons, eliminating the need for scrolling. However, it is worth noting that touch screens can lead to increased postural discomfort during use [59]. Thus, offering a variety of system operation methods might be the most effective way to cater to diverse user needs and preferences. Enhancing usability is paramount not only for optimizing human-computer interactions but also for ensuring the system’s social and practical acceptance [48]. A system’s usability should be of a standard that facilitates effortless task execution by the user. Given that the PRIMS posed challenges for users in performing certain tasks, there is a clear need for usability enhancements.

Another principal finding that came from analyzing the field notes is that the motor examination did not function perfectly or as intended. Similar to any new software system, the PRIMS had its share of technical issues. One of the most frequent issues noted by the RA was that users would perform tests correctly yet the system would fail to score their movements. This is a problem especially when the system does not prompt any corrective feedback yet still informs users that there were not enough valid measurements to score. The software issues caused frustration among the participants and led to multiple usability issues. Usability is tied to functionality, although they are not exactly the same. When a product is not functioning correctly, it ultimately impacts its usability. Thus, ensuring that the system works as it is intended must be a priority for future developers to improve its usability.

Through the analysis of interview transcripts, a prominent theme of appreciation emerged in relation to the PRIMS. Designed with the primary objective of enhancing care for individuals living with PD, the PRIMS was met with significant enthusiasm. Users recognized the immense potential of such a remote monitoring solution, expressing eagerness about its availability. This positive reception underscores the importance of aligning the product’s design with the needs of its intended users. The participants’ commendation of both the system and its mission suggests that the overarching principle of the product is robust. Previous studies have emphasized the pivotal role of consumer perspectives in determining the success of a product [60]. Given this context, such a positive reception indicates a promising trajectory for the future deployment of the PRIMS.

Our last principal finding from this usability study was that our recorded SUS score was 69.7, which failed to meet our acceptable usability score of 70. There are several reasons that could explain why participants felt that the system was not as user-friendly as it should be. Binyamin et al [61] used the SUS to evaluate a learning management system in an educational setting. Their system also failed to meet an acceptable usability score of 70. A distinct aspect of their study was that participants engaged with the system repeatedly throughout a summer term. They observed a direct relationship between the frequency of system use and the SUS score, suggesting that increased familiarity led to improved usability ratings [61]. Drawing from this, it is conceivable that, if participants in our study had interacted with the system over an extended duration, as intended for the PRIMS, the SUS score might have been more favorable due to enhanced user familiarity by the study’s conclusion.

### Severity of Usability Problems

Within the spectrum of presented usability problems, a hierarchical assessment of severity becomes imperative considering factors ranging from potential risks to participant safety to issues causing minor hindrances in task completion. We ranked the issues posing threats to safety and mobility as top priorities to be addressed, followed by those that led to difficulty and frustration, with minor issues that may have slowed participants down being of the least concern.

Foremost among the identified challenges were those associated with mobility constraints, standing out as the most severe due to their inhibiting impact on participants with mobility challenges. Beyond impeding the use of the PRIMS, these challenges pose a risk to participant safety by potentially placing individuals in vulnerable positions. Notably, the constant need for movement to and from the computer for task transitions emerges as a top priority for resolution.

A usability problem of a lesser degree of severity pertains to the system’s sensitivity, specifically in quantifying motor tasks during the PRIMS questionnaire. The requirement for participants to be consistently in a “good position” proved overly demanding, leading to frustration and difficulties, as observed in RA notes and interview transcripts. This underscores the importance of designing systems for individuals with movement disorders to be accommodating to their unique needs. The current state of the PRIMS, requiring users to stay still in certain situations and adopt uncomfortable positions, resulted in skipped tests, increasing the priority of addressing this issue.

Issues that made completion difficult included challenges in navigating using the computer mouse and occasional malfunctions in the motor examination. These concerns, while not as severe as mobility-related issues or the system’s sensitivity, warrant attention as they contribute to user frustration and impact task completion.

Finally, minor difficulties associated with operating the small camera and managing automated voice prompts and written instructions require fine refinements rather than constituting significant hurdles. While not impeding overall system navigation, these issues contributed to slower completion and user frustration. In the hierarchy of severity, they represent areas for enhancement rather than critical concerns requiring immediate attention.

### Implications

This study conducted a comprehensive evaluation of a system specifically tailored for individuals with motor and cognitive conditions, shedding light on critical considerations for the development of technology for this population. First, we advise against the use of desktop applications requiring a computer mouse, scrolling, and intricate clicking, recognizing the potential challenges faced by users. Furthermore, our findings emphasize the superiority of visual instructions over written ones, also suggesting that automated voice prompts should be used judiciously and presented in a friendly manner and offer clear instructions, especially during confirmation processes. To address the mobility challenges commonly faced by this population, systems necessitating movements in front of a camera should minimize the need for multiple adjustments as these can introduce errors. In addition, our study underscores the importance of system flexibility, allowing for a significant margin of error in data capture without imposing the requirement for participants to remain perfectly still during calibration—an often-unattainable feat for those with motor conditions. As we navigate future developments of the PRIMS, these insights will serve as a guide in creating a product that effectively addresses the outlined issues, emphasizing a visually guided interface requiring minimal effort for seamless operation, aligning with the unique needs of our target user base.

### Limitations and Future Investigation

There were several limitations to this study. Our small sample size may not have revealed all the usability issues [62,63] as testing with a small number of participants tends to only reveal the major flaws or glitches in the system. However, our main objective was to uncover the biggest areas of concern rather than identifying every problem associated with the system.

Another limitation to this study was our methodology. Other common qualitative data recording techniques for usability studies include the think-aloud technique [64] and focus groups. The think-aloud technique is the process in which users are encouraged to verbalize their perceptions as they interact with the system [64], which can provide insight into the user experience. Focus groups with study participants following the interviews could have produced richer information. This would have given users the opportunity to compare their ideas and thoughts on the PRIMS. However, while conducting this study, we made a deliberate decision not to use the think-aloud technique. This choice was grounded in our consideration for the unique challenges faced by individuals with PD, particularly those with motor and speech difficulties. The think-aloud technique traditionally involves participants verbalizing their thoughts as they navigate through a system. However, given the potential speech impediments, tremors, and other motor-related challenges associated with PD, we anticipated that asking participants to vocalize their thoughts could introduce unnecessary stress and frustration, and we did not want to pile on any extra cognitive load. To ensure a more comfortable and authentic testing environment for individuals with PD, we opted for direct observation followed by an interview after they were finished using the system, allowing us to carefully note any challenges users encountered as they interacted with the software.

We also acknowledge that other methods, for example, mixed methods research [65], are applicable for usability and user experience research. Mixed methods may allow for a more comprehensive understanding of a user’s experience, which can enable researchers to identify specific usability issues [66]. There are also other scales that we could have used to quantify user satisfaction, for example, the Post-Study System Usability Questionnaire [67]. As our product is in the early stages of development, we opted for a simpler multiple methods study to uncover the major flaws in our system. Future usability studies on the PRIMS can use a mixed methods design to gain a deeper understanding of usability issues.

Considering the intricacies involved in designing systems for users with movement disorders, a promising direction for future research could entail conducting user-centered design studies to tackle the identified usability challenges. This approach, which is advocated by other authors as an effective methodology for achieving a usable product, aims to design products that consider the needs and interests of end users [68-71]. Salinas et al [72] have reviewed the techniques and tools used in the successful redesigns of graphical user interfaces of software products following the user-centered design approach. While some of these techniques align with those used in our evaluation, the key lies in using these techniques throughout the design process instead of solely during product testing. Commonly reported methods of user testing include prototyping, pre- and postdesign interviews, heuristic evaluation, and surveys or questionnaires [72]. Therefore, upcoming research endeavors concerning the redesigned PRIMS should embrace a user-centered design methodology to guarantee the satisfaction of end users’ needs and explore the integration of the aforementioned effective techniques. Moreover, future investigations could concentrate on crafting customizable interface options enabling users to tailor their interaction experience according to their individual capabilities and preferences. This might entail the incorporation of adjustable settings for font size, button layout, and navigation pathways such as voice activation or remote controllers to cater to a diverse range of users.

The current iteration of the PRIMS faces practicality challenges for home use. A more feasible adaptation would necessitate enhanced usability, reduced equipment costs, and minimal space requirements. Substantial updates are imperative to transform the PRIMS into a valuable home-based tool. This entails enhancing user-friendliness, optimizing the product to function seamlessly on common smartphone or tablet cameras, and refining the interface for a user-friendly experience.

It is important to emphasize that a direct score comparison between the PRIMS and a clinician was not conducted in this study. The PRIMS did assign scores to each movement in the motor test using an algorithm developed by PragmaClin Research Inc. However, it is essential to clarify that this study exclusively focused on usability and did not assess the validity of the scoring process. The validation of scoring algorithms remains a subject for future investigations.

### Conclusions

In conclusion, the PRIMS currently exhibits several usability challenges that hinder its efficient use by individuals with PD. For the system to achieve successful implementation and gain broad acceptance, it is imperative to address these identified issues. Feedback from this study is being used to upgrade the PRIMS so that it better aligns with patients’ needs. This study contributes significantly to the growing literature on usability testing, particularly emphasizing design nuances for systems tailored to those with movement disorders. Moving forward, it would be beneficial for future research to explore diverse interaction methods with digital devices, aiming to pinpoint optimal usability practices.

## Appendix

Multimedia Appendix 1 Usability study script.

Multimedia Appendix 2 System usability survey.

Multimedia Appendix 3 Curved grading scale by Lewis and Sauro [55].

## Abbreviations

MDS-UPDRS: Movement Disorder Society–sponsored revision of the Unified Parkinson’s Disease Rating Scale
PD: Parkinson disease
PRIMS: Parkinson’s Remote Interactive Monitoring System
RA: research assistant
SUS: System Usability Scale

## Data Availability

The data sets generated during and analyzed during this study are available in the Memorial University Dataverse-Borealis repository [73].
