# Supplementary material for: Evaluation of the Parkinson’s Remote Interactive Monitoring System in a Clinical Setting: Usability Study
Source: JMIR Hum Factors. 2024 May 24;11:e54145. doi: 10.2196/54145 (PMC11161713; doi:10.2196/54145)
Supplement: Multimedia Appendix 1 [file humanfactors_v11i1e54145_app1.pdf]

Multimedia Appendix 1: Usability Study Script

1) Meet participants in Parking Lot with parking pass
2) Instruct the participant where to sit. Once settled, start the script below.

Before we begin, I have some information for you, and I'm going to read it to make sure that I cover everything. You probably already have a good idea of why we asked you here but let me go over some things again briefly just so we make sure everything makes sense.

So, our study is currently in its early stages of development in Canada so we are looking to just have a small sample of our people with Parkinson's to use the system so we can have a sense of its practicality, accessibility, usefulness, and any problems it might have. We are hoping to do 3 rounds of usability testing throughout the summer to help us refine and improve our system based on user feedback, so we would love to see you again in a few weeks time if you are able.

The system we are testing is the Parkinson's Remote Interactive Monitoring System, or PRIMS for short. It is an integrated system that will monitor, analyze, and rate the severity of a full range of Parkinson's symptoms. It is based off of the Unified Parkinson's Disease Rating Scale, which is the most widely used and verified clinical scale neurologists use to rate disease severity. There are 4 parts to the PRIMS assessment, the system will prompt you and you can just go through them in order if you want. There will be a mix of multiple-choice type questions and also a motor examination where you will be asked to perform certain movements in front of the depth cameras you see in front of you.

When we arrive at the motor examination, I am going to be a little more involved. After each task you perform, I am just going to ask you if everything was clear, for example, one of the tasks you'll have to perform is finger tapping, so when you have moved onto the next task, ill ask if everything was clear, yes or no, and if it wasn't let me know what was not clear, for example, the instructional videos may not explain everything in enough detail or may give you the wrong idea about the task.

Once all the tasks are completed, the software is designed to give a rating based on the all the data collected and put an individual on a Parkinson's rating scale from 0 to 4.

After we have completed everything on the PRIMS system we will end off with a short interview, where I am going to ask you 6 short questions just on things you liked, didn't like, what you would change, what you would like to see added, things like that so keep try to keep those things in mind while working through the system. After that I will give you some time to complete the last short survey. After you have submitted that you are all done.

The entire session will probably take an hour and a half to two hours.

One thing I want to make clear right away is that we're testing the device, not you. You can't do anything wrong here, we are just testing the system for its user friendliness. We would appreciate your honest option, no need to worry about hurting our feelings or anything.

If you have any questions as we go along, just ask them. I may not be able to answer them right away, since we're interested in how people interact with the system when they don't have any help. So, if you ask a question and I don't answer, I am not trying to be rude, its just the study protocol. But if you still have any questions when we're done, I'll try to answer them then, but if you are really stuck then I will

intervene and try to help you out. Also, if you need to take a break at any point, step our or anything just let me know, we are going to be in here for a little while.

You may have noticed the microphone here. With your permission, we're going to record our conversation. The recording will only be used to help us figure out how to improve the system, and it won't be heard by anyone except the people working on this project. All data will be de-identified using number codes, so your name will get a corresponding number which will be used for the remainder of the study, all the recordings will be transcribed and then destroyed so your confidentiality is protected through the entire process.

Last thing I'd like to also stress is that your involvement is completely voluntary, we greatly appreciate that, and you are free to leave at any time you wish.

And have you completed the online consent form?

IF THEY HAVENT COMPLETED THE CONSENT FORM – ok well first things first Ill get you to complete that, you'll find that already opened up in the browser ______, it outlines a lot of what we just talked about so read through it well, when you arrive at the bottom it will ask you to click "I consent" or "I do not consent" then after you complete that it will bring you to a contact information form, after you fill that out, there is also one here that we both sign, and then we will get started with the PRIMS system.

Any questions before we begin?

So, first things first, we need to get you an account so for the username you can just make it your name, and then create your own password, or I have ones already made here, cause its one of these, you need a certain number of numbers, special characters, etc. don't worry about remembering it we are going to keep your user name and password on file for the next time you come in if you wish to do so.

After you log in you can begin the assessment.

As you work through the PRIMS system, we also encourage you to talk to yourself if anything is confusing, or if you do have any questions about something that doesn't make sense to you just vocalize it, because we can include that in our analysis. But again, I probably won't answer or say anything in response.

3) Launch PRIMS assessment tool

START THE RECORDING DEVICE!!!!

4) Arrive at Motor Examination Survey

Now that we are at the motor examination, after each task I am going to ask you if all the instructions are clear, and if not, what was not clear. Also, feel free to adjust the small camera on the tripod on the table as needed, the larger one attached to the computer shouldn't need to be moved at all, but the smaller one probably will have to be so feel free to play with it as needed.

All finished with the PRIMS system. Now I just have a few short questions for you, and after that a short survey. **And feel free to take a break** now if you'd like before the interview.

5) Move into the Qualitative interview.

When we are going thorough the interview feel free to take as long as you want to answer the questions. If you want to skim through the program again before you answer a question, go right ahead, take as much time as you need.

Alright my first questions is:

1- What things did you like most about PRIMS?

2- What things did you like least about PRIMS?

3- Were there things about PRIMS you found confusing or frustrating?

4- What would you like to change about PRIMS?

5- Are there any features you would like to see added to PRIMS?

6- Any overall comments on the PRIMS system?

6) SUS survey

Awesome, that's it for the interview. Last thing is ill get you to switch over to the browser opened on your computer there to complete one last quick survey. And after that we are all done.

Sweet, so you can switch back over to your browser, the survey should already be open there for you, so just click on the "icon of browser" google chrome or edge... and open up the other tab.

To submit the survey just click on the button at the bottom right.

Alright, we are all finished, it was a pleasure to meet you and thanks again for participating! Would you like help finding your way out?
