# Supplementary material for: Evaluation of the Parkinson’s Remote Interactive Monitoring System in a Clinical Setting: Usability Study
Source: JMIR Hum Factors. 2024 May 24;11:e54145. doi: 10.2196/54145 (PMC11161713; doi:10.2196/54145)
Supplement: Multimedia Appendix 2 [file humanfactors_v11i1e54145_app2.pdf]

Multimedia Appendix 2: The System Usability Survey (SUS) as Used by Sauro (2011)

The SUS is a 10-item questionnaire with 5 response options.

1. I think that I would like to use this system frequently.
2. I found the system unnecessarily complex.
3. I thought the system was easy to use.
4. I think that I would need the support of a technical person to be able to use this system.
5. I found the various functions in this system were well integrated.
6. I thought there was too much inconsistency in this system.
7. I would imagine that most people would learn to use this system very quickly.
8. I found the system very cumbersome to use.
9. I felt very confident using the system.
10. I needed to learn a lot of things before I could get going with this system.

The SUS uses the following response format:

Strongly Disagree 1	2	3	4	Strongly Agree 5
○	○	○	○	○

Scoring SUS

- For odd items: subtract one from the user response.
- For even-numbered items: subtract the user responses from 5
- This scales all values from 0 to 4 (with four being the most positive response).
- Add up the converted responses for each user and multiply that total by 2.5. This converts the range of possible values from 0 to 100 instead of from 0 to 40.
