# Supplementary material for: Evaluation of the Parkinson’s Remote Interactive Monitoring System in a Clinical Setting: Usability Study
Source: JMIR Hum Factors. 2024 May 24;11:e54145. doi: 10.2196/54145 (PMC11161713; doi:10.2196/54145)
Supplement: Multimedia Appendix 3 [file humanfactors_v11i1e54145_app3.pdf]

**Multimedia Appendix 3: Lewis and Sauro Curved Grading Scales**

| SUS Score Range | Grade | Percentile Range |
|---|---|---|
| 84.1 - 100 | A + | 96 - 100 |
| 80.8 - 84.0 | A | 90 - 95 |
| 78.9 - 80.7 | A - | 85 - 89 |
| 77.2 - 78.8 | B + | 80 - 84 |
| 74.1 - 77.1 | B | 70 - 79 |
| 72.6 - 74.0 | B - | 65 - 69 |
| 71.1 - 72.5 | C + | 60 - 64 |
| 65.0 - 71.0 | C | 41 - 59 |
| 62.7 - 64.9 | C - | 35 - 40 |
| 51.7 - 62.6 | D | 15 - 34 |
| 0.0 - 51.6 | F | 0 - 14 |
